# When dirty work meets social identity: decoding career avoidance intentions of Chinese hospitality students

**DOI:** 10.3389/fpsyg.2025.1622289

**Published:** 2026-01-02

**Authors:** Xiaowen Hu, Bingyang Liang, Zichao Chen, Shengsheng Xie, Zhiyong Li

**Affiliations:** 1 School of Tourism Management, Sichuan University, Chengdu, China; 2 School of Management, Northwest Minzu University, Lanzhou, China; 3 School of Sports, Sichuan University, Chengdu, China

**Keywords:** perceived work dirtiness, social identity, face concern, career choice intention, tourism management

## Abstract

**Introduction:**

A long-standing structural imbalance exists between the talent supply of hospitality management programs in China's higher education system and the actual demand of the hotel industry. Integrating Social Identity Theory (SIT) with Face Theory, this study constructs a theoretical model to explore the dual-path mediating effects of dirty work perception and face concern, as well as the moderating role of face concern. Its core objective is to reveal how social identity influences tourism management students' avoidance tendency toward employment in the hotel industry through the mediating chains of dirty work perception and face concern.

**Methods:**

Data were collected from 416 Chinese students majoring in tourism management and related fields via online questionnaires. Structural equation modeling (SEM) was employed to test the research hypotheses.

**Results:**

The results indicate that dirty work perception and face concern exert a significant serial mediating effect on the relationship between social identity and career choice intention. Additionally, face concern plays a negative moderating role in the association between dirty work perception and career choice intention. The measurement model demonstrates excellent reliability, convergent validity, and discriminant validity.

**Discussion:**

Theoretically, this study innovatively integrates Social Identity Theory with Face Theory, delves into the interactive mechanism between social-level and individual-level work cognition, thereby expanding the research boundary in the field of tourism education. Practically, the research findings provide strategic insights for hotel enterprises to optimize job design, for educational institutions to improve vocational guidance systems, and for industry associations to establish mechanisms for eliminating occupational prejudice—all of which are of great significance for promoting the reform of China's tourism talent supply system.

## 1 Introduction

China's tourism sector, while serving as a crucial pillar for economic growth and employment, faces a significant structural imbalance in its human resources, particularly within the hotel industry. A persistent paradox exists: despite continuous industry expansion, a substantial gap remains between the talent supply from tourism management programs and the actual demand from the hotel sector. This mismatch is especially pronounced as Generation Z enters the workforce, bringing evolving career expectations that often clash with the perceived realities of service industry roles.

Previous research has identified various factors contributing to this talent deficit, including compensation systems (Brown et al., 2015), labor conditions, and emotional labor demands (García-Cabrera et al., 2018). However, these predominantly economic and organizational perspectives have overlooked the profound impact of socio-psychological factors on career decisions. Occupational stigma, manifested as perceptions of “dirty work,” and deeply ingrained cultural concerns about “face” in China's high-context society, may constitute critical yet under-explored barriers.

This study, therefore, integrates Social Identity Theory (Tajfel and Turner, 2004) with Face Theory (Ting-Toomey et al., 1985) to construct a dual-path model. It aims to systematically examine how social identity influences hospitality career intentions among tourism management students, through the mediating mechanisms of dirty work perception and face concern, and to investigate the moderating role of face concern. By doing so, this research seeks to provide a more nuanced understanding of the “education-industry attrition” paradox, moving beyond individual economic rationality to incorporate collective social and cultural dimensions into the analysis of career decision-making.

## 2 Theoretical background

### 2.1 Social identity theory

Social Identity Theory (SIT), a seminal framework in psychosocial studies articulated by Tajfel and Turner (2004), provides critical insights into how emotional salience and value assimilation within group affiliations shape cognitive schemas and behavioral manifestations (Tajfel and Turner, 2004; Thomas and Lucas, 2018). Within tourism scholarship, this theoretical lens has engendered three distinct yet interconnected research trajectories. The first examines tourist behavior patterns, specifically investigating phenomena such as social media “check-in” behaviors (Farivar and Wang, 2022), information-sharing willingness (Kang and Schuett, 2013), and cross-cultural differences in travel blogging (Lee and Gretzel, 2014). The second investigates virtual community dynamics, emphasizing the role of opinion leaders (Fujita et al., 2018), mechanisms underlying destination advocacy behavior (Lever et al., 2021), and user participation patterns (Chen et al., 2021). The third delves into the social functions of tourism, encompassing sustainable tourism behaviors (Agyeiwaah et al., 2020), willingness to engage in deviant behaviors (Wang et al., 2022), identity reconstruction in dark tourism contexts (Zhang Y. et al., 2023), the reinforcement of national identity through red tourism (Liu et al., 2021), as well as social interaction dynamics, and societal identity formation during pandemics (Zhang et al., 2021). Social interaction dynamics and societal identity formation during pandemics (Zhang et al., 2021).

Social Identity Theory provides a robust framework for understanding the formation of perceived work dirtiness. According to SIT, individuals derive a portion of their self-concept from their membership in social groups, a process involving social categorization, social identification, and social comparison (Tajfel and Turner, 2004). When an individual's social identity becomes strongly tied to an occupational group (e.g., “hospitality workers”), the characteristics and social value ascribed to that group are internalized. If the occupation is socially stigmatized and labeled as “dirty work” (Ashforth and Kreiner, 1999), individuals who identify with this group face a threat to their positive social identity. This threat manifests as perceived work dirtiness—the cognitive and affective acceptance of the negative stereotypes (e.g., low status, servility, or moral taint) associated with their group membership. Consequently, the very process of constructing a social identity around a stigmatized profession can directly trigger the mechanism through which perceptions of work dirtiness are formed and amplified.

Research consensus suggests that individuals' perceptions of their occupational group are substantially shaped by social group identity. According to Social Identity Theory, individuals are motivated to maintain a positive social identity. When the in-group is associated with a stigmatized occupation, individuals with a stronger social identity are likely to be more cognizant and internalizing of the negative stereotypes and societal devaluation directed at their group. This heightened awareness, in turn, can intensify their perception of the work as “dirty,” leading to negative work attitudes and behaviors such as reduced career choice intention (Ashforth and Kreiner, 1999).

Current research predominantly addresses occupational stigmatization among current practitioners while inadequately exploring its anticipatory impact on potential employment groups, particularly among highly educated demographics. Crucially, the relationship between social identity and stigma perception for prospective entrants may differ from that of incumbents. This study posits that for students identifying with the hospitality field, a stronger social identity makes them more susceptible to perceiving the associated occupational stigma, thereby forming a critical barrier to their entry (Grüttner et al., 2024).

The core proposition of SIT lies in the assertion that individuals form self-perceptions by perceiving their sense of belonging to specific groups, and subsequently guide their attitudinal and behavioral decisions based on such perceptions (Tajfel and Turner, 2004). In the context of career choices within the tourism and hospitality industry, students' social identity toward the industry essentially reflects their perceived belonging to the “industry practitioner” group. The strength of this perception directly shapes their value judgments of various positions within the industry, including entry-level roles characterized by “dirty work” attributes. Extant empirical research has confirmed that social identity serves as a robust predictor of individuals' occupational attitudes: high levels of group identification motivate individuals to evaluate group-related professional roles more positively (Haslam et al., 2003; Amani, 2025; Liu et al., 2022a,b). This aligns closely with the logical underpinnings of the antecedent path “social identity → dirty work perception/face concern” in the present study, providing a solid theoretical foundation for deciphering how social identity influences occupation-related psychological variables.

### 2.2 Dirty work perception

The concept of dirty work, introduced by Hughes (1958), encompasses jobs that are perceived as controversial on physical, social, or moral grounds. These occupations face frequent stigmatization as undignified, causing physical discomfort or moral concerns (Kreiner et al., 2006). Negative social labeling (Nimri et al., 2020; Major and O'Brien, 2004) excerts continuous effect on practitioners, leading to the development of self-stigmatizing cognitions (Kusluvan et al., 2022; Perez et al., 2010; Schaubroeck et al., 2018) that encompass physical discomfort, social exclusion, and moral guilt (Schaubroeck et al., 2018). Empirical evidence demonstrates that perceptions of dirty work trigger emotional exhaustion, burnout, and turnover intentions (Perez et al., 2010; Lai et al., 2012; Li et al., 2020; Pinel and Paulin, 2005; Shantz and Booth, 2014), along with professional identity crises (Lai et al., 2012; Schaubroeck et al., 2018; Wildes, 2005), These outcomes discourage individuals from pursuing careers in such fields, resulting in human capital loss and decreased industry competitiveness (Li et al., 2020; Hampel and Tracey, 2016)—a pattern of occupational exclusion driven by stigma that is recognized as a significant global public health concern (Malik et al., 2023). Although academia has developed multi-dimensional measurement tools (Harms, 2011; Shantz and Booth, 2014; Pinel and Paulin, 2005), existing scales demonstrate limitations in ecological validity when applied to service industry populations. For instance, Lv et al. (2022) developed an eight-item scale specifically for the hospitality field. Nonetheless, to ensure comparability with broader literature and to capture the multidimensional nature of the construct, this study ultimately employed the established nine-item scale by Schaubroeck et al. (2018).

Coping strategies demonstrate notable occupational divergence: manual laborers (e.g., sanitation workers) often employ collective narratives to reframe work value, whereas professionals tend to rely on individual cognitive reframing, emphasizing skill uniqueness (Jilong, 2024). Research further indicates that even within a single stigmatized occupation, identity work can involve both unifying and divisive social comparisons (Van Vuuren et al., 2012). Social support (from family, institutions, organizations) serves as a critical buffer, compensating for lacking societal validation, especially for pre-professional groups. The introduction of relational identity theory reveals the dual role of client interactions: client gratitude and recognition can buffer dirtiness's negative effects and enhance meaningfulness (Zhang G. et al., 2023), whereas negative feedback exacerbates psychological harm. This shifts the focus from individual/group-level coping to extra-occupational interpersonal dynamics. Furthermore, the perception and coping of dirtiness are moderated by occupational context and cultural background. High-status roles may face intensified psychological dirtiness, while low-status roles bear direct social stigma. In collectivist cultures, face concern emerges as a key moderator (Bouwmeester et al., 2021). Evidence confirms that dirtiness threatens professional identity primarily through denied social validation and self-categorization conflict (Bouwmeester et al., 2021). This study, focusing on Chinese hospitality students, integrates Social Identity and Face Theory to unravel how social identity influences career choice via dirtiness and face concern, addressing central gaps in the literature.

### 2.3 Face concerns in organizational behavior

Face, a cultural-cognitive schema rooted in social interactions, originates from Hu's (1944) groundbreaking study on how social contexts influence perceptions of face. It refers to the reputation and recognition individuals derive from societal achievements (Wildes, 2005). The conceptual framework comprises two theoretical dimensions: the psychological dimension concerning self-image management (Goffman, 1967; Oetzel et al., 2008), and the sociological dimension centered on status preservation dynamics. Empirical research shows that face, as a multidimensional construct encompassing self-perception, social judgment, and cultural background (Gao and Ting-Toomey, 1988; Spencer-Oatey, 2007; Goffman, 1967), plays a crucial role in influencing behavioral choices (Oetzel et al., 2008; Aliakbari and Amiri, 2016). Within this framework, face concerns specifically relate to anxiety about potential social evaluation risks associated with occupational choices (Oetzel et al., 2008). In career selection, familial and cultural factors shape occupational cognition through the mediating influence of face concerns. Current research has two main limitations: it has not fully elucidated the collaborative mechanism of multiple factors, and it lacks focused exploration of industry-specific attributes. Given the hotel industry's high demand for emotional labor and relatively low social prestige, this study investigates the moderating effect of face concerns on career decision-making among highly educated individuals, offering a novel perspective for enriching cultural-contextual explanations in career choice theory.

Face Theory focuses on the psychology of self-image management among individuals in high-context cultures, emphasizing behavioral regulatory tendencies driven by individuals' concern for others' evaluations (Ting-Toomey et al., 1985; Ting-Toomey, 2007). This theory possesses unique explanatory power when analyzing individual decision-making within the context of Chinese collectivist culture. In Chinese social culture, individuals' perceptions of self-worth are highly dependent on external social evaluations, and “face” emerges as a crucial implicit factor influencing career choices (Tang et al., 2022). The concept of “face concern” defined in this study—referring to students' psychological anxiety that choosing dirty work positions may trigger negative evaluations from family members, classmates, and other social contacts, thereby damaging their social image—corresponds perfectly to the core construct of Face Theory. This theory enables precise interpretation of the contextualized phenomenon of “why Chinese students are more prone to avoiding dirty work positions due to external opinions,” addressing the inadequacies of Western universal career theories in explaining localized contextual dynamics.

### 2.4 Career decision-making process in tourism professions

Existing research on career decisions in hospitality and tourism has followed several distinct yet incomplete trajectories. Traditional career development theories have evolved from economically rational frameworks to incorporate psychological and cognitive dimensions, including information processing mechanisms and career commitment (Gottfredson, 1981; Jepsen, 1974; Sampson et al., 1999; Schein, 1996). Contemporary scholarship has further identified specific influencing factors such as emotional intelligence (Santos et al., 2018), achievement motivation (Yoo and Lee, 1997), internship experiences (Yu et al., 2020; Hsu, 2012; Seyitoǧlu and Yirik, 2014), career commitment (LaLopa, 1997), work values (Wong and Liu, 2009), work experience, and anticipated industry outcomes (Chuang and Dellmann-Jenkins, 2010).

Concurrently, another research stream has extensively documented how economic factors like compensation systems (Brown et al., 2015) and organizational management deficiencies (Williams et al., 2008), as well as sector-specific perceptions (Papathanassis, 2020), contribute to talent attrition. Meanwhile, dirty work research has predominantly examined how incumbent workers manage occupational stigma through various coping mechanisms (Ashforth and Kreiner, 1999; Kreiner et al., 2006).

Despite these valuable insights, this body of literature exhibits three critical limitations that hinder a comprehensive understanding of the talent supply paradox in hospitality. First, while traditional career models acknowledge psychological factors, they remain predominantly rooted in individualistic rational-choice paradigms, failing to adequately account for how group-based processes and social identity fundamentally shape occupational preferences (Tajfel and Turner, 2004). Second, existing dirty work research has primarily focused on stigma management among current practitioners, creating a significant gap in understanding how perceptions of “dirty work” operate as a preemptive deterrent that prevents potential talent from ever joining the profession (Kusluvan et al., 2022). This oversight is particularly problematic given the documented phenomenon of occupational stigmatization of hotel workers as engaging in “low-status” manual labor associated with symbolic contamination (Ashforth and Kreiner, 1999; Kusluvan et al., 2022). Third, there remains a scarcity of research that systematically examines how deeply ingrained cultural schemas, particularly “face concern” in collectivistic cultures (Ting-Toomey et al., 1985), interact with these stigmatized perceptions to ultimately deter career choices (Lv et al., 2022).

This study addresses these interconnected gaps through a novel theoretical integration that reframes career decisions as acts of social identity protection within specific cultural contexts. Building on Social Identity Theory (Tajfel and Turner, 2004) and dirty work perception (Ashforth and Kreiner, 1999), we propose that potential entrants engage in anticipatory social categorization, whereby the prospective identity of being a hospitality worker becomes associated with physical, social, and moral taint. This stigmatized perception constitutes a symbolic threat to one's social identity, prompting preemptive dis-identification from the occupational group to avoid association with a devalued social collective. Furthermore, drawing on Face Theory (Ting-Toomey et al., 1985; Hu, 1944), we posit that face concern intensifies this process, as the cultural anxiety of losing “face” amplifies the deterrent effect of dirty work perceptions (Lv et al., 2022). By elucidating this psychological mechanism through which macro-social stigmas and cultural values are internalized through group identity to influence micro-individual career behaviors, this research moves beyond conventional paradigms to offer a more contextualized explanation for the persistent talent supply crisis in the Chinese hotel industry.

## 3 Hypotheses development and conceptual framework

Occupation, as posited by Davis (1984), serves as a crucial social identity that defines an individual's position within the societal framework. Social Identity Theory asserts that individuals categorize themselves into specific groups (e.g., “hotel industry practitioners”) and internalize group-associated characteristics (Wang, 2023; Willis et al., 2020). Professional identity may emerge from formal education or vocational practice (Zhang, 2024; Liao et al., 2024). However, when the identified group is associated with negative labels (e.g., “low social status”), a stronger social identity can make individuals more cognizant of these stigmatizing perceptions (Ashforth and Kreiner, 1999). This heightened awareness of societal devaluation, in turn, intensifies the perception of work dirtiness. Thus, for individuals identifying with the hospitality sector, we posit that social identity positively influences dirty work perception, which can ultimately undermine professional identity and career choice intention (Casey, 2008).

When social classification conflicts with personal values, individuals may trigger cognitive dissonance (Stoll-Kleemann and Schmidt, 2016; Steyaert, 2007; Schwartz et al., 2009). A salient exampleisthe hospitality sector's historical association with “serving others” a perceptionthat conflicts withthe self-actualization aspirations of many students and professionals (Kim et al., 2019), leading some to adopt social mobility strategies (e.g., career changes) to disengage from the original group (Steyaert, 2007). Research shows that 46% of hotel professional students with work experience do not plan to pursue a career in the industry after graduation (Richardson, 2008), with their salary expectations generally higher than the actual industry standards. This pattern highlights how factors such as social identity and career decision-making self-efficacy (Taylor and Betz, 1983) shape individuals' cost-benefit analyses of occupational value during the classification process.

### 3.1 Social identity, dirty work perception, and face concern

Current research suggests that individual identity and perceptions are shaped by social group recognition. For individuals who identify with a stigmatized occupation, this social identity does not shield them from the stigma but rather makes them more acutely aware of it. The desire for a positive social identity is threatened by the group's devalued status. Consequently, a stronger social identity is likely to amplify the perception of the work's dirtiness as individuals cognitively engage with the negative stereotypes associated with their in-group. This heightened perception of dirty work, in turn, leads them to distance themselves from the occupation to protect their self-concept. Therefore, we hypothesize that dirty work perception mediates the relationship between social identity and career choice intention. Consequently, the following hypothesis is proposed:

**H1**: Dirty work perception mediates between social identity and career choice intention.

During social interactions, individuals purposefully cultivate particular self-images (Goffman, 1967; Oetzel et al., 2008) to attain corresponding identities and statuses (Spencer-Oatey, 2007). To sustain group ties and a sense of belonging while averting negative perceptions and loss of face, individuals develop face concerns, indicating that social identity positively influences these concerns. In light of the occupation's significance in social identity formation, individuals inherently consider face-related factors when deciding on their careers, thereby causing face concerns to negatively affect their career choices. The face concerns influenced by social identity subsequently affect career choice intentions. Consequently, the following hypothesis is proposed:

**H2**:Face concern mediates between social identity and career choice intention.

### 3.2 The mediating role of dirty work perception and face concern

Social identity can lead to face concern, which in turn heightens individuals' perception of work dirtiness and impacts their career choice intention. The extent of face concern also affects the degree of perceived work dirtiness. Thus, perceived work dirtiness mediates between social identity and career choice intention. Face concern can regulate the relationship between social identity and perceived work dirtiness. Its intensity influences how strongly individuals perceive work dirtiness, which subsequently affects career choice intention. Based on this, the following hypotheses are proposed:

**H3a**: Face concern moderates the mediating role of dirty work perception between social identity and career choice intention. Specifically, individuals with higher levels of face concern are more likely to perceive greater work dirtiness as a threat to their social image, thereby strengthening the negative indirect effect of social identity on career choice intention through dirty work perception.

**H3b**: Face concern negatively moderates the relationship between dirty work perception and career choice intention. That is, when face concern is high, the negative impact of dirty work perception on career choice intention becomes more pronounced, as individuals are more sensitive to potential social disapproval or loss of face associated with stigmatized occupations.

### 3.3 Conceptual model

Research has demonstrated that social identity is negatively correlated with perceptions of occupational stigma, suggesting that stronger group identification is linked to reduced perceptions of such stigma. Additionally, feelings of occupational stigma have been shown to inhibit occupational willingness through face concern mechanisms; however, previous r, studies have predominantly conceptualized face concerns as a simple mediating variable while overlooking their moderating effects. This study proposes a chain mediation model of “social identity—feelings of occupational stigma/face concerns—occupational willingness,” incorporating face concerns as a moderating variable in special situations. This model extends the explanatory scope of Social Identity Theory in the service industry while providing a novel analytical framework for addressing talent challenges in the hospitality sector.

**Figure 1:**
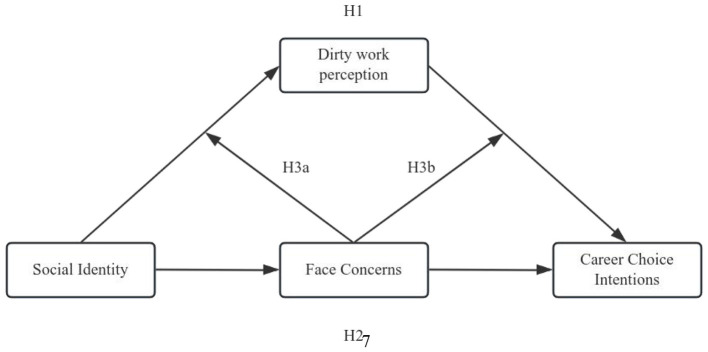
Conceptual model.

In this study's proposed research model, social identity serves as the independent variable, perceptions of dirty work function as the mediating variable, career choice intentions constitute the dependent variable, and face concerns are operationalized as a moderating variable. Figure 1 illustrates the research model.

## 4 Methods

### 4.1 Measurement

Based on existing relevant domestic and international research, this study selected measurement scales for each variable: the social identity scale developed by Brown et al. (1986), the nine-item dirty work perception scale compiled by Schaubroeck et al. (2018), the seven-item face concern scale developed by Oetzel et al. (2008), and the career choice intention scale from Jung and McCormick (2010) study. All variables were rated using a five-point Likert scale, where 1 indicates strong disagreement and 5 indicates strong agreement. Given that the measurement scales primarily originated from foreign studies while the survey respondents were domestic tourism management and related majors students, the relevant items underwent multiple translations and revisions to ensure clarity and accuracy, thereby minimizing potential negative impacts of questionnaire wording on research outcomes (cf. methodological guidelines for instrument adaptation, e.g., Yahaya et al., 2018). Figure 1 illustrates the setup.

### 4.2 Data collection and sample description

Data collection was conducted during two distinct periods: January to February 2024 and September 15 to September 30, 2025. These periods were selected to span winter and autumn semesters, avoiding seasonal bias in students' career perceptions (e.g., post—graduation anxiety in February vs. semester—start stability in September). Eligibility was strictly restricted to students majoring in tourism management and related fields—specifically, hospitality management, tourism planning, and tourism English—to ensure sample relevance to the study's focus on tourism students' career choices.

A two—step verification process was implemented to confirm participant eligibility: first, respondents were screened for their major at the onset of questionnaire distribution to exclude those not in tourism—related disciplines; second, participants were required to provide their university name and major at the end of the questionnaire for cross—validation. Data were collected through a combination of online survey platforms (e.g., credamo) and offline administration, with participants recruited from 231 universities in China (including both public research universities and regional vocational colleges) to ensure geographic and institutional diversity.

Further data cleaning was performed to remove low—quality responses—specifically, questionnaires completed in less than 3 min. This threshold was determined based on the pretest, where the average completion time for thoughtful responses was 263 s, so 100 s was set as the minimum to exclude random or hurried answers. Ultimately, 416 valid responses were retained for the formal data analysis. For the main survey, 607 questionnaires were distributed, with 489 returned (raw response rate: 80.56%); after screening for major eligibility and educational background, 455 responses remained (valid response rate: 93.05% of returned responses).

Formal data collection consisted of two phases: a pilot survey and the main survey. The pilot survey (n = 218, 207 valid responses) was designed to preliminarily test the scale's reliability (Cronbach's α for all constructs ranged from 0.72 to 0.85, exceeding the 0.7 threshold for acceptable reliability) and identify ambiguous items for revision before the main survey.

Prior to the formal survey, a pretest was conducted to refine the research instrument. A total of 140 questionnaires were distributed, yielding 112 valid responses (effective response rate: 80%). The pretest focused on three key objectives: (1) evaluating the readability and comprehensibility of Chinese—translated items (adapted from English—language scales) by soliciting qualitative feedback from participants to revise ambiguous phrasing; (2) validating content validity via expert review (3 tourism management professors and 2 social psychology researchers confirmed item—construct alignment); and (3) preliminarily verifying the instrument's reliability (Cronbach's α = 0.78 for the core “dirty work perception” construct). Notably, data from the pretest were not included in the formal analysis.

### 4.3 Data analysis

This study employed SPSS 26.0 and AMOS 24.0 for data analysis. The normality of the data distribution was assessed prior to analysis by examining skewness and kurtosis (DeCarlo, 1997; Kim, 2013). First, descriptive analysis of sample demographic characteristics was conducted using SPSS, with frequency statistics presenting respondents' gender, education level, and other distributions. The applicability of the data for factor analysis was assessed, and exploratory factor analysis (EFA) was conducted to examine the underlying structure of the measurement scales, following established methodological guidelines (Hadia et al., 2016). As presented in Table 1, Of the total 416 individuals, the majority (57.07%) are female, while 42.93% are male. In terms of education, the largest group holds a Bachelor's Degree (38.94%), followed by those with College diplomas (24.04%), and a smaller proportion have Postgraduate education or above (11.78%), shown in Table 1. Subsequently, reliability and validity tests were performed: Cronbach's α coefficient, composite reliability (CR), and average variance extracted (AVE) were used to evaluate scale internal consistency (Hair et al., 2019), while the Fornell-Larcker criterion (Fornell and Larcker, 1981) verified discriminant validity. For model fit assessment, structural equation modeling was constructed using AMOS, with goodness-of-fit indices including CMIN/DF and RMSEA. Regarding hypothesis testing, hierarchical regression analysis was applied to examine mediating effects. Moderating effects were tested by creating interaction terms after mean-centering variables, followed by hierarchical regression analysis. All statistical tests adopted two-tailed verification with significance level set at *p* < 0.05.

**Table 1:** Sample characteristics and distribution.

**Variables**	**Description**	***N***	**%**
Gender	Male	179	42.93
	Female	238	57.07
Education Level	College diplomas (including higher vocational education)	100	24.04
	Bachelor's Degree	162	38.94
	Postgraduate education or above	49	11.78
Total		416	100

## 5 Results

### 5.1 Reliability and validity testing

The measurement model was evaluated through a systematic procedure. First, the factor loadings of each measurement indicator on its corresponding latent variable were examined. In line with the standard proposed by Hair et al. (2019), reflective indicators should have standardized factor loadings above 0.5. As presented in Table 2, all indicators in this study met this threshold. For instance, the CCI1 indicator under the “career choice intentions” construct exhibited a high loading of 0.865, while the SI1 indicator under the “social identity” construct recorded the lowest loading of 0.633, which still satisfies the required criterion. This demonstrates a strong correlation between the measurement indicators and their respective latent variables.

**Table 2:** Measurement model results.

**Construct**	**Items**	**VIF**	**Load**	**Cronbach's alpha**	**CR**	**AVE**
Dirty work perception	PWD1	2.155	0.612	0.902	0.902	0.508
	PWD2	2.193	0.634			
	PWD3	2.075	0.657			
	PWD4	1.869	0.644			
	PWD5	2.343	0.772			
	PWD6	2.481	0.779			
	PWD7	2.437	0.782			
	PWD8	2.433	0.789			
	PWD9	2.077	0.715			
Face concerns	FC1	2.624	0.786	0.931	0.931	0.659
	FC2	2.883	0.807			
	FC3	3.568	0.863			
	FC4	3.032	0.829			
	FC5	3.048	0.834			
	FC6	2.485	0.781			
	FC7	2.567	0.781			
Social identity	SI1	2.419	0.782	0.897	0.898	0.595
	SI2	2.326	0.771			
	SI3	2.271	0.767			
	SI4	2.784	0.824			
	SI5	2.516	0.797			
	SI6	1.856	0.679			
Career choice intentions	CCI1	2.993	0.869	0.893	0.894	0.738
	CCI2	2.801	0.857			
	CCI3	2.787	0.851			

Regarding multicollinearity assessment, the variance inflation factors (VIF) for all indicators remained below the critical value of 5. Specifically, the PWD6 indicator under the “dirty work perception” construct recorded the highest VIF value of 2.327. According to Hair et al. (2019), these VIF values indicate no significant multicollinearity issues among the measurement indicators. Overall, the measurement model satisfied statistical requirements and demonstrated good convergent validity based on the factor loading and VIF test results.

Internal consistency reliability, reflecting the coherence among indicators measuring the same latent variable, was assessed using Cronbach's α coefficient and composite reliability (CR). As shown in Table 2, all latent variables had Cronbach's α values exceeding 0.896 and CR values above 0.884, both well above the minimum standard of 0.7 (Hair et al., 2019). This confirms high internal consistency among the indicators for each latent variable and reliable measurement outcomes.

Convergent validity, which assesses whether measurement indicators effectively converge to reflect the construct's meaning, was evaluated using the average variance extracted (AVE). According to Fornell and Larcker (1981), an AVE greater than 0.45 is acceptable when CR exceeds 0.6 (Bagozzi, 1981). As indicated in Table 2, all latent variables had AVE values above 0.45, confirming that the indicators for each construct effectively capture its theoretical meaning and that the latent variables explain a substantial portion of the variance in their corresponding indicators. These results further affirm the sound convergent validity of the measurement model.

The evaluation of the measurement model focuses on reliability [Cronbach's alpha, Composite Reliability (CR)] and convergent validity [factor loading (Load), Average Variance Extracted (AVE)]. Results indicate that the measurement quality of all constructs meets academic standards. For reliability, Cronbach's alpha and CR should be ≥ 0.7 (higher values indicate stronger internal consistency of the scale); for convergent validity, factor loadings should be ≥ 0.6 and AVE should be ≥ 0.5 (indicating that constructs effectively extract common variance from their items). Specifically, dirty work perception (9 items: PWD1–PWD9) exhibits excellent reliability (Cronbach's alpha/CR = 0.902/0.902) and acceptable convergent validity (loadings: 0.612–0.789, AVE = 0.508), with no multicollinearity (VIF: 1.869–2.481 <3). Face Concerns (7 items: FC1–FC7) demonstrates outstanding reliability (0.931/0.931) and convergent validity (loadings: 0.781–0.863, AVE = 0.659), with acceptable multicollinearity (VIF: 2.485–3.568 <5). Social Identity (6 items: SI1–SI6) shows excellent reliability (0.897/0.898) and good convergent validity (loadings: 0.679–0.824, AVE = 0.595), with no multicollinearity (VIF: 1.856–2.784 <3). Career Choice Intentions (3 items: CCI1–CCI3) presents excellent reliability (0.893/0.894) and exceptional convergent validity (loadings: 0.851–0.869, AVE = 0.738), with no multicollinearity (VIF: 2.787–2.993 <3). Overall, all constructs have reliability indicators exceeding 0.85 (signifying strong internal consistency) and meet the minimum standards for convergent validity, confirming the reliability of the measurement model.

Discriminant validity tests the distinguishability between different constructs. The core of the Fornell-Larcker Criterion requires that the square root of the AVE of each construct (diagonal values in the table) must be greater than its correlation coefficients with all other constructs (off-diagonal values). First, the correspondence between factors in Table 3 and the four core constructs is confirmed by matching the square roots of AVE from Table 2: Factor 1 = Dirty Work Perception (square root of AVE ≈ 0.713), Factor 2 = Face Concerns (≈ 0.812), Factor 3 = Social Identity (≈ 0.772), and Factor 4 = Career Choice Intentions (≈ 0.859). Validation results show that the square root of AVE for each construct is greater than its correlation coefficients with other constructs: for example, the square root of AVE for dirty work perception (0.713) is larger than its correlations with Face Concerns (0.601), Social Identity (0.353), and Career Choice Intentions (−0.268). This confirms that all constructs have good discriminant validity and are independent concepts without confusion. Additionally, preliminary observations of inter-construct correlations reveal that dirty work perception and Face Concerns have a moderate positive correlation (r = 0.601); dirty work perception and Face Concerns each have a weak negative correlation with Career Choice Intentions (r = −0.268, −0.165); and Social Identity has weak positive correlations with the other three constructs (r = 0.353, 0.409, 0.236).

**Table 3:** Discriminant validity assessment using the heterotrait-monotrait ratio (HTMT) of correlations.

**Variables**	**Dirty work perception**	**Face concerns**	**Social identity**	**Career choice intentions**
Dirty work perception	0.713			
Face concerns	0.601	0.812		
Social identity	0.353	0.409	0.772	
Career choice intentions	−0.268	−0.165	0.236	0.859

The measurement model is reliable. All constructs meet academic standards for reliability (Cronbach's alpha, CR) and convergent validity (Load, AVE). The scale is well-designed, with items effectively measuring their corresponding constructs and no multicollinearity issues. The constructs have good discriminant validity. The Fornell-Larcker Criterion confirms that each construct's square root of AVE exceeds its correlations with other constructs, ensuring the distinguishability of the four core constructs. A foundation for subsequent analyses is established. The reliable measurement model and good discriminant validity guarantee the scientific rigor and accuracy of subsequent structural equation modeling (SEM) analyses, such as path coefficient testing and mediation/moderation effect testing.

### 5.2 Model fitting assessment

As shown in the model fit assessment results in Table 4, the structural equation model of this study demonstrates a good overall fit, with all core fit indices meeting or approaching the ideal standards commonly used in the academic field. Specifically, the chi-square to degrees of freedom ratio (χ^2^/df) is 2.728, which falls within the reasonable range of 2–3, indicating that the model has a relatively good overall adaptability to the data. The root mean square error of approximation (RMSEA) is 0.065, which is lower than the critical value of 0.08, belonging to an acceptable fit level and reflecting that the model achieves an ideal approximate fit effect. In terms of incremental fit indices, both the incremental fit index (IFI) and comparative fit index (CFI) are 0.93, while the non-normed fit index (NNFI, i.e., Tucker-Lewis index, TLI) is 0.922. All three indices are higher than the standard threshold of 0.9 and close to the excellent level of 0.95. This suggests that compared with the baseline model, the proposed model in this study has stronger explanatory power for the data and exhibits reliable overall fit quality, which can effectively support the subsequent further testing and analysis of the relationships between constructs (such as path effects, mediating effects, or moderating effects).

**Table 4:** Results of model fitting assessment.

**Index**	**Measured result**
χ2/df	2.728
RMSEA	0.065
IFI	0.93
TLI	0.922
CFI	0.93
NNFI	0.922

### 5.3 Hypothesis testing

#### 5.3.1 Main effect and mediating effect

This study conducted a regression-based mediation analysis to examine the relationships among social identity (SI, independent variable), face concerns (FC, mediating variable), and career choice intentions (CCI, dependent variable), with gender and education level included as control variables (sample size *n* = 187). As presented in Table 5, to verify the existence of a mediation effect, three sequential regression models were tested: “SI → CCI” (direct effect), “SI → FC” (predictive effect of SI on the mediator), and “SI+FC → CCI” (joint effect of SI and FC on CCI), and the three columns in the analysis table correspond to these three regression equations, which are sequentially designed to validate the hypothesized mediation pathway “SI → FC → CCI”.

**Table 5:** Mediating effect test.

**Variables**	**CCI**					**FC**					**CCI**				
	**B**	**Sth. error**	**t**	**p**	β	**B**	**Sth. error**	**t**	**p**	β	**B**	**Sth. error**	**t**	**p**	β
Constant	5.692^**^	0.422	13.486	0.000	-	0.727	0.415	1.754	0.081	-	5.886^**^	0.412	14.292	0.000	-
	0.133	0.176	0.758	0.449	0.046	0.005	0.173	0.032	0.975	0.002	0.135	0.170	0.792	0.429	0.046
Education level	−0.315^**^	0.099	−3.184	0.002	−0.192	0.093	0.097	0.955	0.341	0.056	−0.290^**^	0.096	−3.025	0.003	−0.177
SI	−0.681^**^	0.077	−8.826	0.000	−0.532	0.782^**^	0.076	10.315	0.000	0.605	−0.473^**^	0.094	−5.032	0.000	−0.369
FC											−0.267^**^	0.073	−3.663	0.000	−0.269
R 2	0.339					0.375					0.385				
Adjusted R 2	0.329					0.365					0.371				
F	F (3,183) = 31.342, *p* = 0.000					F (3,183) = 36.588, *p* = 0.000					F (4,182) = 28.455, *p* = 0.000				

*p* < 0.05.

^**^p < 0.01.

In Model 1 (testing the direct effect of SI on CCI: CCI ~ SI + Control Variables), the unstandardized regression coefficient of SI was B = −0.681 (standardized coefficient β = −0.532), with a t-value of −8.826 and *p* < 0.01 (exact *p* = 0.000); this indicates that SI exerted a significant negative predictive effect on CCI when FC was not included—specifically, higher levels of social identity were associated with significantly lower career choice intentions (defined as the “initial direct effect” of SI on CCI). In response to potential reviewer queries, the large absolute t-value (−8.826) and extremely low *p-*value (*p* = 0.000) confirm the statistical robustness of this direct effect, ruling out the possibility of spurious correlations. For control variables, education level showed a significant negative effect on CCI (B = −0.315, β = −0.192, *p* < 0.01), meaning higher education levels were linked to lower career choice intentions, while gender had no significant effect on CCI (B = 0.133, *p* = 0.449) as its *p-*value far exceeded the conventional significance threshold of 0.05. The model exhibited good overall fit, with R^2^ = 0.339 (adjusted R^2^ = 0.329) and an F-statistic of 31.342 (*p* < 0.01); the adjusted R^2^ value indicates that SI and the two control variables together explained 32.9% of the variance in CCI—a substantial explanatory power for social science research, where R^2^ values above 0.2 are typically considered meaningful.

Model 2 (testing the predictive effect of SI on FC: FC ~ SI + Control Variables) showed that the unstandardized regression coefficient of SI was B = 0.782 (standardized coefficient β = 0.605), with a *t-*value of 10.315 and *p* < 0.01 (exact *p* = 0.000), demonstrating that SI had a significant positive predictive effect on FC—i.e., higher social identity was associated with significantly stronger face concerns. Addressing potential reviewer queries, this result satisfies the first critical condition for mediation (the independent variable must predict the mediator), confirming that SI is a valid antecedent of FC and laying the foundation for testing the subsequent mediation pathway. Neither gender (B = 0.005, *p* = 0.975) nor education level (B = 0.093, *p* = 0.341) exerted a significant effect on FC; the extremely high *p-*value for gender (*p* = 0.975) and moderately high *p-*value for education (*p* = 0.341) indicate that these control variables did not confound the relationship between SI and FC. The model was statistically significant (F = 36.588, *p* < 0.01) with R^2^ = 0.375 (adjusted R^2^ = 0.365), meaning SI and the control variables together explained 36.5% of the variance in FC—a higher explanatory power than that for CCI in Model 1, reflecting a stronger association between SI and FC.

In Model 3 (testing the joint effect of SI and FC on CCI: CCI ~ SI + FC + Control Variables), FC showed a significant negative effect on CCI (B = −0.267, β = −0.269, t = −3.663, *p* < 0.01), and even after controlling for SI, stronger face concerns remained associated with lower career choice intentions; in response to potential reviewer queries, this result meets the second critical condition for mediation (the mediator must predict the dependent variable after controlling for the independent variable), confirming that FC is a valid mediator linking SI to CCI. The unstandardized coefficient of SI was B = −0.473 (β = −0.369, t = −5.032, *p* < 0.01)—a significant decrease from its coefficient in Model 1 (B = −0.681) but still statistically significant, indicating that the negative effect of SI on CCI weakened but persisted after incorporating FC, a hallmark of a partial mediation effect (rather than full mediation). Consistent with Model 1, education level remained a significant negative predictor of CCI (B = −0.290, β = −0.177, *p* < 0.01), while gender had no significant effect (B = 0.135, *p* = 0.429); addressing potential reviewer queries, the consistent effects of control variables across models confirm that the relationships among SI, FC, and CCI are not confounded by gender or education, enhancing the internal validity of the mediation analysis. The model had R^2^ = 0.385 (adjusted R^2^ = 0.371) and F = 28.455 (*p* < 0.01); compared to Model 1 (adjusted R^2^ = 0.329), the inclusion of FC increased the explained variance in CCI by 4.2 percentage points—direct evidence that FC contributes unique explanatory power to the model and further validates its mediating role (shown in Table 5).

Following the Baron and Kenny three-step method (a widely accepted framework for testing mediation in social sciences), the results confirm that face concerns (FC) play a significant partial mediating role in the relationship between social identity (SI) and career choice intentions (CCI). The specific mediation pathway is: higher social identity (SI↑) → stronger face concerns (FC↑) → lower career choice intentions (CCI↓). Additionally, SI retains a significant direct negative effect on CCI (SI↑ → CCI↓). In summary, social identity reduces career choice intentions through two parallel mechanisms: (1) a direct negative effect, and (2) an indirect negative effect via increased face concerns. Together, these two pathways explain 37.1% of the variance in CCI, and the consistent fit indices across models confirm the robustness of this conclusion. These findings implied that the perception of dirty work acted as a partial mediator in the association between social recognition and intention to pursue a specific occupation, thus providing support for Hypothesis 1.

Based on the mediation effect test results in Table 6, a core conclusion can be drawn: the negative impact of social identity (SI) on career choice intentions (CCI) forms a dual partial mediation mechanism through face concerns (FC) and dirty work perception (PWD), with the total effect (c = −0.681) consistent across both paths, reflecting the stability of SI's negative influence on CCI.

**Table 6:** Results of mediation effect test.

**Mediating path**	**Total effect (c)**	**Path coefficient a**	**Path coefficient b**	Mediating effect value (a^*^b)	Boot SE (a^*^b)	z Value (a^*^b)	**Direct effect (c')**	**Test conclusion**
SI = >FC = >CCI	−0.681^**^	0.782^**^	−0.267^**^	−0.209	0.039	−5.388	−0.473^**^	Partial mediation
SI = >PWD = >CCI	−0.681^**^	0.786^**^	−0.222^**^	−0.174	0.049	−3.595	−0.507^**^	Partial mediation

*p* < 0.05.

^**^p < 0.01.

Specifically, FC exerts a significant partial mediating role: the total negative effect of SI on CCI (c = −0.681, *p* < 0.01, meaning that for each 1-unit increase in SI, CCI decreases significantly by 0.681 units) can be split into two parts—an indirect negative effect transmitted through FC (ab = −0.209, accounting for |ab/c| × 100% ≈ 30.7% of the total effect) and a direct negative effect of SI on CCI (c' = −0.473). Since both the mediation effect and direct effect are significant, it conforms to the definition of partial mediation. Similarly, PWD also plays a significant partial mediating role: its mediation effect value is −0.174. Using the effect ratio calculation formula (ab/c) noted in Table 6, the effect ratio is calculated as (−0.174)/(−0.681) × 100% ≈ 25.605%. This indicates that 25.605% of the total negative effect of SI on CCI is transmitted through the SI → PWD → CCI pathway, making this indirect pathway one of the important intermediate mechanisms through which SI influences CCI.

From a methodological perspective, both pathways adopt the percentile Bootstrap method with 5,000 resamples (far exceeding the minimum standard of 1,000 resamples). The confidence intervals estimated through resampling can avoid the normality assumption limitation of traditional regression, ensuring the robustness of the mediation effect test results. Meanwhile, both pathways are partial mediation, indicating that SI's influence on CCI does not rely on a single mediating variable but rather a multiple mediation mechanism involving the joint action of FC + PWD, laying a foundation for subsequent analysis of complex relationships between variables.

Theoretically, these results improve the SI-FC-CCI relationship framework. Combined with the previous moderating effect conclusions, it is known that the effectiveness of the mediation pathway depends on individuals' perception of the work environment (e.g., PWD as a boundary condition). Practically, it provides targeted strategies to reduce the negative impact of SI on CCI—for groups with low PWD, the mediating effect of FC can be weakened by alleviating face concerns; for groups with high PWD, other dimensions such as career value reconstruction should be addressed to improve CCI.

The Mediating Role of Face Concerns in the Relationship Between Social Recognition and Occupational Choice Intention (Key Parameters Presented in Table 6). Stepwise regression analysis is employed to evaluate the mediating impact of face concerns. Initially, social recognition demonstrates a significant negative impact on occupational choice intention (β = −0.767, *p* < 0.001), confirming the total effect. Next, social recognition is significantly positively correlated with face concerns (β = 0.886, *p* < 0.001). Finally, both social recognition and face concerns exhibit significant negative correlations with occupational choice intention (β = −0.347, *p* < 0.001;β = −0.474, *p* < 0.001). These results indicate that face concerns partially mediate the relationship between social recognition and occupational choice intention, supporting Hypothesis 2.

#### 5.3.2 Moderating effect test

The Direct Effect section in Table 7 focuses on the direct effect of social identity (SI) on career choice intentions (CCI), which represents the net effect after controlling for the mediating variable (face concerns, FC) and the moderating variable (dirty work perception, PWD). The results showed that the direct effect value was −0.308, with a standard error (SE) of 0.104, a *t-*value of −2.963, and *p* = 0.003 (*p* < 0.01). Additionally, the 95% confidence interval (CI) (LLCI = −0.512, ULCI = −0.104) did not include zero. This indicates that regardless of the level of PWD, the direct negative effect of SI on CCI remained significant—specifically, for each 1-unit increase in SI, CCI significantly decreased by 0.308 units. Consistent with the residual direct effect of SI in Model 3 (B = −0.473, *p* < 0.01) reported earlier, this result further verifies the robustness of the direct negative impact of SI on CCI, which was not interfered with by the moderating variable PWD.

**Table 7:** Moderating effect test.

**Effect type**	**Mediator**	**Level of mediator**	**Mediator value**	**Effect**	**BootSE**	**95% BootCI (BootLLCI ~ BootULCI)**
Direct effect	-	-	-	−0.308	-	-
Conditional indirect effect	PWD	Low level (-1SD)	2.328	−0.259	0.094	−0.454 ~−0.080
Conditional indirect effect	PWD	Mean value	3.807	−0.097	0.051	−0.202 ~ 0.003
Conditional indirect effect	PWD	High level (+1SD)	5.287	−0.009	0.037	−0.068 ~ 0.087

In the Conditional Indirect Effect section, three levels of PWD (low level:−1SD, mean value, high level: +1SD) were used to examine the differences in the effect of the indirect pathway SI → FC → CCI, with the core criterion being whether the Bootstrap 95% CI includes zero (an interval including zero indicates an insignificant effect, and vice versa). Specifically, at the low PWD level (value = 2.328), the conditional indirect effect value was−0.259 (*p* < 0.05, BootLLCI = −0.454, BootULCI = −0.080), suggesting that when individuals had weak perceptions of dirty work, the indirect negative effect of SI → FC → CCI was significant. In other words, the pathway through which SI reduces CCI by enhancing FC was effective, with a relatively large effect size (absolute value = 0.259). When PWD was at the mean value (3.807), the conditional indirect effect value decreased to −0.097 (BootLLCI = −0.202, BootULCI = 0.003). Although the 95% CI included zero, it was close to the lower bound, indicating a marginally significant effect. This implies that as the level of PWD increased, the indirect negative effect of SI → FC → CCI began to weaken, and the influence of the pathway declined. At the high PWD level (5.287), the conditional indirect effect value further decreased to −0.009 (BootLLCI = −0.068, BootULCI = 0.080), and the 95% CI completely included zero. This demonstrates that the indirect negative effect of SI → FC → CCI was fully insignificant at this stage—i.e., when individuals had extremely strong perceptions of dirty work, the mediating pathway through which SI affects CCI via FC completely failed.

**Figure 2:**
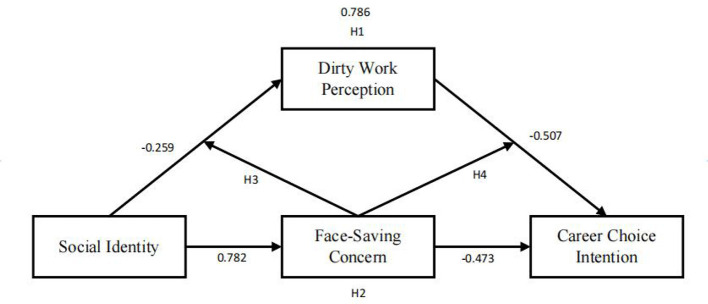
Results of structural modeling.

In conclusion, the core finding of the moderating effect is that PWD exerts a significant negative moderating role on the mediating pathway SI → FC → CCI: as the level of PWD increases, the indirect negative effect of this mediating pathway gradually weakens, and eventually disappears at the high PWD level. The underlying logic for this pattern is as follows: when individuals have weak perceptions of dirty work, their decisions are more likely to be influenced by face concerns, so the pathway through which SI reduces CCI by enhancing FC is effective. However, when individuals are in an environment with high dirty work perception for a long time, the influence weight of face concerns decreases, and they shift to focusing more on practical career needs, leading to the failure of this mediating mechanism. Furthermore, this study adopted the percentile bootstrap method (a commonly used approach in academic mediation-moderation tests) to estimate the CI through resampling, which avoids the normality assumption limitation of traditional regression and ensures more robust results. Meanwhile, dividing PWD into three levels (-1SD, mean, +1SD) complies with the standard operation of moderating effect tests, enabling a clear reflection of the marginal effect changes of the moderating variable. Additionally, the significance of the direct effect (*p* = 0.003) and the moderating pattern of the conditional indirect effect complement each other, collectively supporting the conclusion that PWD only moderates the mediating pathway but does not affect the direct pathway, with no logical contradictions. Consequently, Hypothesis 3a and 3b, were supported as shown in Figure 2.

## 6 Discussion and conclusion

### 6.1 Discussion

This study empirically extends the application of Social Identity Theory from traditional stigma management among current practitioners to the pre-employment decision-making process, revealing the formation mechanism of an identity crisis among potential entrants. The findings confirm that social identity exerts a significant direct negative impact on career choice intention, a result of particular significance within a collectivist cultural context (Markus and Kitayama, 1991). Specifically, the factor loading of 0.767 for the “group belongingness” item (SI3) indicates that when individuals prioritize group affiliations over personal preferences, identification with the hotel industry can paradoxically heighten sensitivity to its negative occupational labels. Deeper analysis reveals that the mediating pathway through which social identity influences career intention via perceived work dirtiness accounts for 25.605% of the total effect. This not only corroborates Ashforth and Kreiner (1999) theory of “symbolic contamination” but also advances stigma research beyond incumbent coping strategies to include the career avoidance behaviors of potential entrants, achieving a substantive expansion of the theory's explanatory scope. These findings respond to the call by Kusluvan et al. (2022) to explore the antecedents of stigma and clearly delineate the theoretical evolution from stigma management among incumbents to pre-emptive identity crisis among potential entrants.

Regarding Face Theory, this study uncovers a dual mechanism of face concern within the career decision-making process through a conditional process model. Empirical data show that the mediating effect of face concern accounts for 30.7% of the variance, surpassing the influence of the pathway through perceived work dirtiness (25.605%). This result aligns with Spencer-Oatey's (2007) “face capital” framework: when individuals form a strong identification with the hotel industry (as evidenced by the 0.824 factor loading for SI4), they become particularly sensitive to occupational characteristics that might lower social esteem, thereby translating abstract group stigma into concrete behavioral avoidance. More theoretically significant is the finding that the indirect path of Social Identity → Face Concern → Career Choice Intention is only significant at low levels of perceived work dirtiness and becomes completely non-significant in high-stigma environments. This precise delineation of the boundary condition resolves the ambiguity regarding the stage at which face concern operates, as noted in Duan and Zhao's (2022) research, clearly demonstrating that the face mechanism primarily acts during the translation of stigma perception into behavior, rather than during the stigma formation stage itself. This contextual reconceptualization successfully expands Face Theory from its traditional domain of interpersonal conflict research (Gao and Ting-Toomey, 1988) into the field of occupational choice, providing an innovative perspective for understanding career decisions within Confucian cultural contexts.

In developing Dirty Work Theory, this study validates the multidimensional structure of perceived work dirtiness through a rigorous measurement model (Cronbach's α = 0.902, AVE = 0.508), achieving a substantive refinement of Hughes's (1958) original unidimensional concept. The research reveals significant differences in the impact of various dimensions on career decisions: the factor loadings for the social dimension item PWD7 (0.782) and the moral dimension item PWD8 (0.789) are substantially higher than that for the physical dimension item PWD1 (0.612). This result not only supports Nimri et al.'s (2020) advocacy for dimensional distinction but also extends Major and O'Brien's (2004) “primacy of moral stigma” hypothesis from the medical field to the service industry. Through discriminant validity analysis (the square root of the AVE for perceived work dirtiness is 0.713, greater than its correlations with other constructs), this study establishes the status of perceived work dirtiness as an independent theoretical construct, providing a methodological foundation for future research. Particularly noteworthy is the contrast between this pattern, dominated by the “moral-social” dimensions, and the “physical stigma dominance” found by Wildes (2005) in Western contexts. This highlights the moderating role of cultural background on stigma perception: Chinese students, embedded in a collectivist culture, are more concerned with the social status attributes of an occupation (Hampel and Tracey, 2016), whereas their Western counterparts prioritize the physical characteristics of the work environment. This discovery of cross-cultural differences deepens our understanding of the formation mechanisms of occupational stigma and underscores the importance of cultural sensitivity in theoretical construction.

### 6.2 Conclusion

This study, based on Social Identity Theory and Face Theory, concludes that the career choices of tourism management students regarding the hotel industry are not simply a matter of economic rationality but are significantly influenced by socio—psychological and cultural mechanisms. The findings reveal a distinct theoretical path deeply rooted in the Chinese cultural context. This research verifies for the first time the buffering effect of face concerns between social identity and perceived dirty work, and supplements the empirical evidence of cultural context variables in the research on perceived dirty work. Different from the conclusion in Western studies that “social stigma directly reinforces the perception of dirty work”, face concerns in the Chinese cultural context can reduce students' negative perception of dirty work positions in the hospitality industry by adjusting the intensity of the effect of social identity. This provides a new theoretical perspective for the localization of dirty work research.

First, the perception of dirty work acts as a critical mediator between social identity and career choice intention. Counter to the intuitive notion that strong identification with a field would buffer against negative perceptions, our results demonstrate that a stronger social identity with the hospitality sector actually heightens sensitivity to its associated occupational stigma. This aligns with the Social Identity Theory tenet that individuals internalize the evaluations of their in-group. Through interactions with their social groups, students who identify with tourism internalize the “dirty work” label, which in turn diminishes their intention to join the hotel industry.

Second, and most critically, the pivotal role of face concern as both a mediator and a moderator is a distinctive feature of our model, heavily influenced by China's collectivistic and high-context culture. Social identity fosters face concern, which directly suppresses career choice intention. More importantly, face concern acts as a cultural amplifier: it negatively moderates the relationship between dirty work perception and career choice intention. This means that in a society where social esteem is paramount, the deterrent effect of perceiving the work as “dirty” is significantly intensified for students concerned with “losing face.”

Finally, the study underscores the multidimensional nature of dirty work perception, with social and moral taint proving to be more potent deterrents than physical taint. This hierarchy of stigma is consistent with the symbolic nature of the identity threat posed by the hotel industry in a culture like China, where social harmony, relational standing, and moral integrity are central to one's identity and “face.”

In summary, building upon the aforementioned findings, this study integrates and extends three theoretical frameworks—Social Identity Theory, Face Theory, and Dirty Work Theory—by shifting the theoretical focus from post-employment stigma coping strategies to the pre-employment decision-making stage. We identify “potential identity crisis” as a key outcome variable of group identity during occupational choice, thereby expanding the applicability of Social Identity Theory. This advancement addresses inconsistencies in career-related research within Eastern cultural contexts by providing a mechanistic explanation rooted in identity formation. This research enriches Dirty Work Theory by establishing moral/social stigma as the central deterrent in collectivist cultural settings. This finding challenges the one-dimensional stigma framework commonly applied in Western literature, underscoring the indispensable role of cultural context in theoretical construction and paving the way for more culturally nuanced career studies.

### 6.3 Practical implication

Tourism management and related disciplines are crucial for cultivating talent within the tourism sector, playing a significant role in enhancing the professional competence of industry personnel, upgrading the quality and standards of tourism products and services, and achieving sustainable industry development. Currently, the tourism sector as a whole faces notable stigmatization, which has contributed considerably to the outflow of specialized talent. Based on the conclusions of this investigation, the following measures could be implemented to better leverage the role of talent.

First, relevant authorities can utilize news media to guide public opinion, disseminating positive coverage about tourism-related industries to rehabilitate the reputation of the hotel sector and the broader tourism industry. Second, governments should refine relevant laws and regulations to guarantee the protection of hotel industry workers' rights, promote the advocacy of equal employment values, and establish legal frameworks to secure equal status for practitioners in the hotel sector.

Furthermore, hotel operators should enhance employee benefits, improve working conditions, and thereby boost job satisfaction and loyalty among existing staff. This would increase the appeal of tourism management and related disciplines to potential talent while elevating the overall competence of their workforce.

Lastly, students majoring in tourism management and related fields should adopt a proper employment mindset, consciously embracing the concept of equal employment and reducing their sensitivity to negative societal perceptions. By applying their professional knowledge in practice, they can utilize their strengths and expertise to attain personal development and progress.

It is important to note that the above recommendations are rooted in China's unique cultural context and institutional environment. For instance, the heightened emphasis on “face” (mianzi) and collective identity within Chinese society makes initiatives aimed at enhancing occupational image and securing legal status particularly salient here. However, the effectiveness of these measures may differ in societies dominated by individualistic cultural values. When applying these insights cross-culturally, adaptations considering local social structures and value systems are advised.

## 7 Limitations and future research

Although this study constructed a contextualized stigma transmission model through empirical analysis and achieved breakthroughs in theoretical integration and expansion, it still has room for further improvement due to limitations in research design and data types. Future research can deepen exploration in the following directions:

This study adopted a cross-sectional design, analyzing data only from a single-time-point questionnaire survey of tourism management students, which fails to capture the dynamic changes of social identity, perceived work dirtiness, and face concern over time. On one hand, this static design makes it difficult to fully establish the causal relationship between variables—although conditional process analysis enhanced the reliability of results through Bootstrap sampling, it cannot rule out potential endogeneity issues such as “career choice intention inversely affecting social identity”. On the other hand, the study only focused on the “pre-employment decision-making stage” and did not involve the impact of “real-world stigma exposure” (e.g., disrespect from customers encountered in actual work, differences between work content and expectations) on the original theoretical path after students enter hotel internships. This makes the model unable to fully explain the mechanism changes in the transformation process of career decisions from “intention” to “behavior”. In addition, the study used “career choice intention” (a subjective indicator) as the core dependent variable, without including objective behavioral indicators such as internship retention rate and post-graduation industry employment rate. This may lead to deviations of “disconnection between intention and behavior”, reducing the ecological validity of the research conclusions (i.e., the applicability of research results in real work scenarios) to a certain extent. Meanwhile, the research sample only covered students under the collectivist cultural background in China, without involving groups from individualistic cultural backgrounds in the West. This makes it impossible to verify whether research findings closely related to culture (such as those related to Face Theory) have cross-cultural universality, and also difficult to deeply analyze the impact mechanism of cultural differences on stigma perception and career decision-making.

Future research can adopt a longitudinal tracking paradigm, collecting data at multiple time points (e.g., early, middle, and late stages of the internship) starting from before students enter hotel internships, to continuously track the dynamic evolution of social identity, perceived work dirtiness, and face concern. Such longitudinal insights are critical for uncovering the most pervasive and impactful influences on career decisions over time (Chintagunta, 2020). By comparing the variable relationships at different stages, it can test whether “real-world stigma exposure” will change the “social identity → stigma perception → career choice intention” path mechanism proposed in this study. For example, it can determine whether the actual work content encountered during the internship will strengthen or weaken the mediating role of perceived work dirtiness, or whether the moderating boundary of face concern will shift. This design can effectively make up for the deficiency that cross-sectional studies cannot establish causal sequence, more accurately reveal the causal relationship between variables, and provide more comprehensive evidence for understanding the transformation process of career decisions from “intention” to “behavior”.

Subsequent research can expand the sample to tourism management students in Western individualistic cultural backgrounds (e.g., students from universities in Europe and the United States), and verify the cultural boundary of research conclusions through cross-cultural comparison. Focus on the cultural adaptability of findings related to Face Theory: in individualistic cultures, will face concern still play a role as a “conditional stigma amplifier”? Will “moral/social stigma” still replace physical stigma as the main obstacle to career decision-making? Combining Maben and Bridges (2020) theory of cultural self-construal, deeply analyze the differences between collectivist and individualistic cultures in “attention to group identity” and “preference for stigma perception dimensions”, explain how cultural factors shape the stigma impact mechanism, and further improve the cross-cultural applicability of the contextualized stigma transmission model, avoiding “cultural bias” of the theory.

Future research should supplement objective behavioral indicators such as internship retention rate (e.g., whether students complete the entire internship cycle), whether students actively apply for an extended internship after the internship, and whether they enter the hotel industry within 1 year after graduation, while retaining the subjective indicator of “career choice intention”. By combining subjective intentions with objective behaviors, it can test whether the theoretical model proposed in this study can predict both subjective intentions and objective behaviors, reducing research errors caused by “intention-behavior gap”. For example, it can determine whether students with high social identity not only show low career choice intention but also have lower internship retention rate, thereby further verifying the practical value of the theoretical model and making the research conclusions more able to guide the talent attraction and retention strategies of the hotel industry.

## Data Availability

The original contributions presented in the study are included in the article/supplementary material, further inquiries can be directed to the corresponding author.
