# Correction: When dirty work meets social identity: decoding career avoidance intentions of Chinese hospitality students

**DOI:** 10.3389/fpsyg.2026.1864406

**Published:** 2026-07-13

**Authors:** Xiaowen Hu, Bingyang Liang, Zichao Chen, Shengsheng Xie, Zhiyong Li

**Affiliations:** 1 School of Tourism Management, Sichuan University, Chengdu, China; 2 School of Management, Northwest Minzu University, Lanzhou, China; 3 School of Sports, Sichuan University, Chengdu, China

**Keywords:** perceived work dirtiness, social identity, face concern, career choice intention, tourism management

Affiliation [^1^School of Tourism Management, Sichuan University, Chengdu, China, ^2^School of Management, Northwest Minzu University, Lanzhou, China] was omitted for author [Xiaowen Hu]. This affiliation has now been added for author [Xiaowen Hu].

Affiliation [^1^School of Tourism Management, Sichuan University, Chengdu, China] was omitted for author [Zhiyong Li]. This affiliation has now been added for author [Zhiyong Li].

The funder [Fundamental Research Funds for the Central Universities of Northwest Minzu University (Grant No. 31920240034), Gansu Provincial Higher Education Teaching Achievement Cultivation Project (Grant No. 2024GSJXCGPY-81), Undergraduate Talent Training Quality Improvement Project of Northwest Minzu University (Grant No. 2025ZDJG-02), and National Social Science Fund of China (Grant No.22BMZ06)] was erroneously omitted. The corrected Funding statements appears below.

## Funding

The author(s) declared that financial support was received for this work and/or its publication. This study was funded by Fundamental Research Funds for the Central Universities of Northwest Minzu University (Grant No. 31920240034), Gansu Provincial Higher Education Teaching Achievement Cultivation Project (Grant No. 2024GSJXCGPY-81), Undergraduate Talent Training Quality Improvement Project of Northwest Minzu University (Grant No. 2025ZDJG-02), and National Social Science Fund of China (Grant No.22BMZ06).

The original version of this article has been updated.

